# One-year survivor of adult alveolar rhabdomyosarcoma of the maxillary sinus with orbital extension: Case report

**DOI:** 10.1097/MD.0000000000011866

**Published:** 2018-08-21

**Authors:** Jin-Ho Joo, Ji Sang Han, Shin-Myeong Choi, In-Ki Park, Jae-Ho Shin

**Affiliations:** a Department of Ophthalmology, Kyung Hee University Hospital at Gangdong, Kyung Hee University School of Medicine; b Department of Ophthalmology, Kangbuk Samsung Hospital, College of Medicine, Sungkyun kwan University; c Department of Ophthalmology, Kyung Hee University Hospital, Kyung Hee University School of Medicine, Seoul, Korea.

**Keywords:** adult rhabdomyosarcoma, alveolar variants, one-year survival, orbit tumor

## Abstract

**Introduction:**

Rhabdomyosarcoma is uncommon in adults. Adult and maxillary rhabomyosarcoma with direct orbital extension has been rarely reported. To our knowledge, there is no reported case about adult patient with alveolar maxillary rhabdomyosarcoma and orbital extension survived 1 year with intact ocular function.

**Case presentation:**

A 21-year-old female presented with protrusion of the right eye and an obstructed nasal passage for the past month. Her symptoms were not relieved by oral antibiotic or irrigation. She was referred to our clinic. Computed tomography and magnetic resonance imaging showed a large homogenous well-enhanced mass with surrounding bony erosion and remodeling. The mass extended to the ipsilateral nasal cavity and orbit. Endoscopic biopsy of the nasal cavity confirmed alveolar rhabdomyosarcoma. The maxillary mass was excised using the Caldwell–Luc approach, and the orbital mass was excised using a transconjuctival and transcaruncular approach. A systemic work-up confirmed ipsilateral lymph node metastasis. The patient received 6 cycles of chemotherapy with vincristine, dactinomycin, and cyclophosphamide, as well as 5120 cGy radiotherapy. Her ocular function was intact 1 year after treatment, and magnetic resonance imaging showed complete regression of the tumor.

**Conclusion:**

Rhabdomyosarcoma, which is usually an aggressive malignancy, should be considered in the differential diagnosis of a rapidly growing orbital mass. Aggressive treatment, including surgery, chemotherapy, and radiation therapy, can increase local remission rates and improve the prognosis.

## 1 Introduction

Rhabdomyosarcoma is the most common primary orbital malignancy in children <15 years of age, accounting for approximately 5% of childhood malignancies.^[1]^ Rhabdomyosarcoma is uncommon in adults. The predilection sites of adult rhabdomyosarcoma are the extremities but the predilection sites in children with rhabdomyosarcoma are the head and neck area. Head and neck sites only account for 24% of adult rhabdomyosarcoma cases.^[2]^ Moreover, cases involving the orbit are extremely rare.

The prognosis of adult rhabdomyosarcoma is poor. More than 60% of adult patients have regional or distant metastasis at the initial diagnosis.^[3]^ It is very difficult or impossible to surgically excise a mass within the maxillary area. When a tumor occurs in this region, it is usually more extensive locally or has metastasized distantly at the time of diagnosis. The 5-year survival rate is ≤8% in cases of head and neck rhabdomyosarcoma.^[4]^ Herein, we report the case of a 1-year survivor of adult alveolar rhabdomyosarcoma of the maxillary sinus with orbital extension.

Written informed consent was obtained from the patient for this case report. Ethical approval was obtained by the Institutional Review Board of Kyung Hee University Hospital at Gangdong (KHU-2010-07-39).

## 2 Case report

**Figure 1:**
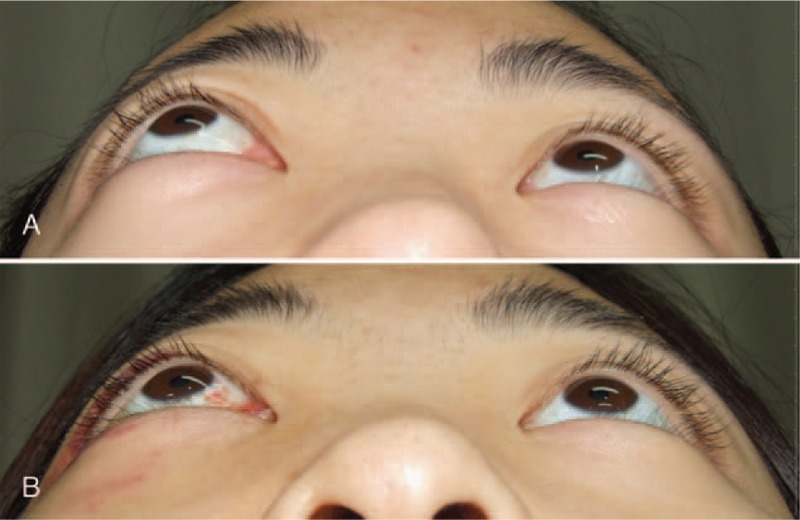
The photographics proptosis of the patient's right eye before (A) and after (B) surgery. Before surgery, her right eye became 3 mm proptosis. And eye proptosis was decreased after surgery.

**Figure 2:**
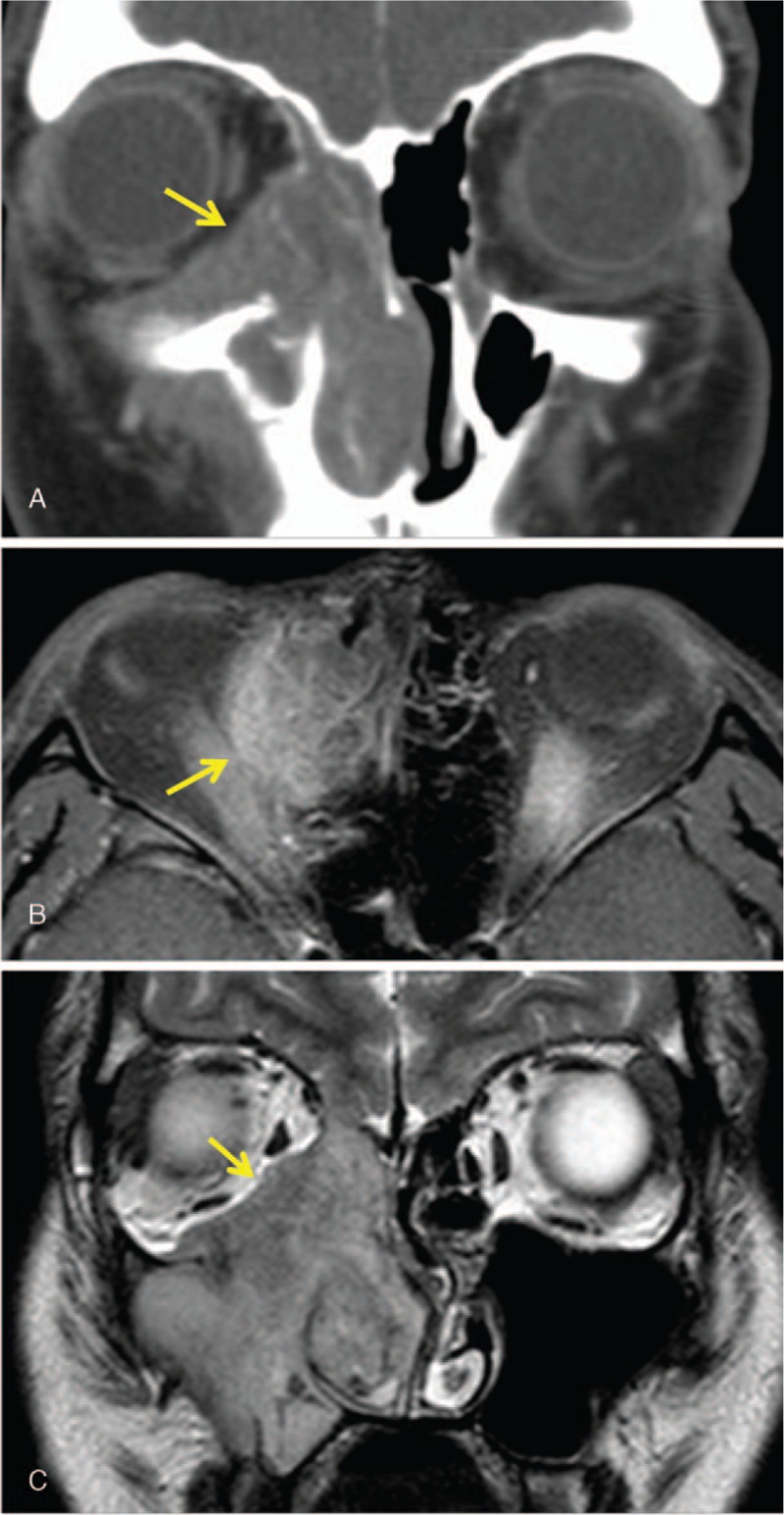
Coronal computed tomography scan (A), axial (B) and coronal (C) magnetic resonance imaging showing a large homogenous well-enhanced mass (6.4 × 4.7 cm) with surrounding bony erosion and remodeling.

**Figure 3:**
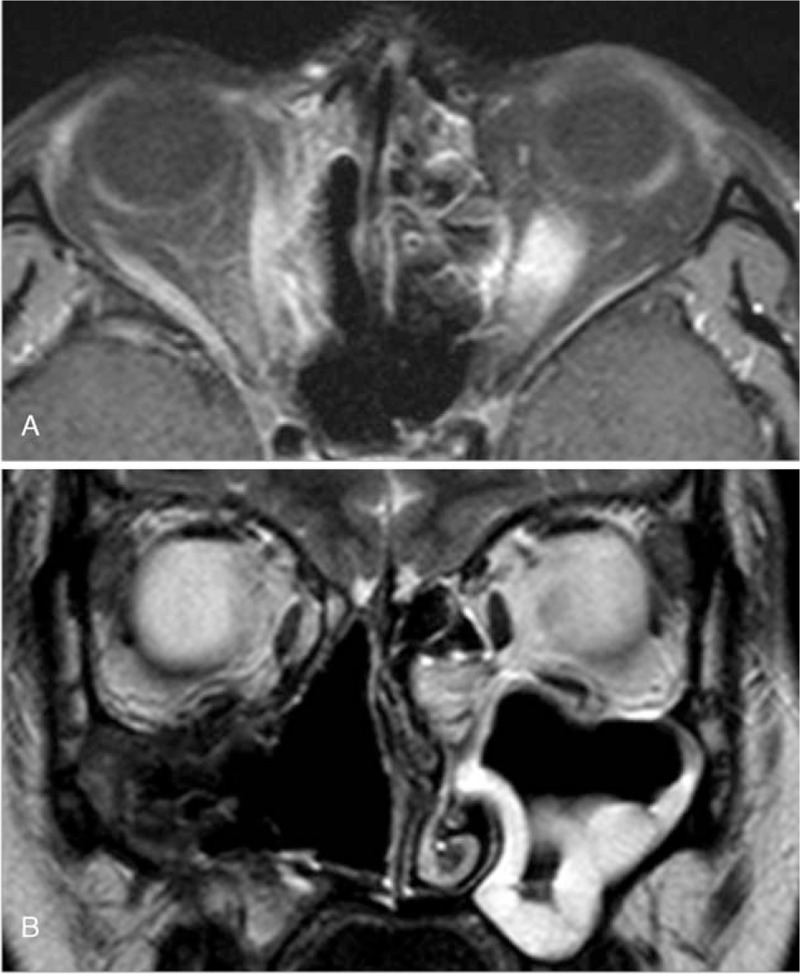
Axial (A) and coronal (B) magnetic resonance imaging showing postoperative right maxillary mucosal wall thickening and left maxillary sinusitis 1 year after treatment.

A 21-year-old female without specific medical history presented with a protruding right eye and an obstructed nasal passage of 1-month duration. The patient was diagnosed with sinusitis at another clinic and was prescribed oral antibiotics. Her symptoms were not relieved by the antibiotic treatment or nasal irrigation. She was referred to our clinic, and we performed a diagnostic work-up. Corrected visual acuity was 1.0 in both eyes. Hertel exophthalmometry revealed 3-mm proptosis (Fig. 1A). The extraocular muscle was intact. No specific findings were observed in the anterior or posterior segments. Computed tomography and magnetic resonance imaging (MRI) showed a large homogenous well-enhanced mass with surrounding bony erosion and remodeling (Fig. 2). The mass had extended to the nasal cavity and right orbit. Regional neck lymph node involvement was observed. A fiber-optic endoscopic biopsy of the nasal cavity confirmed alveolar rhabdomyosarcoma. The immunohistochemical analysis was positive for desmin, myeloperoxidase, and CD56, consistent with the diagnosis. As neck lymph node metastasis was suspected; surgical debulking, chemotherapy, and radiation therapy were scheduled. The maxillary and nasal cavity mass was excised using the Caldwell–Luc approach, and the orbital mass was excised through a transconjuctival incision in the inferior fornix followed by a caruncular incision. Complete tumor removal was difficult because the tumor contained the orbital wall and was located near the optic canal. The excised mass was pathologically confirmed as alveolar rhabdomyosarcoma. The right eye proptosis was relieved after surgery (Fig. 1B). Ultrasonography-guided fine needle aspiration of a neck lymph node confirmed malignancy of the tumor. The Intergroup Rhabdomyosarcoma Study Group (IRSG) postsurgical staging was group 3. The patient underwent 6 cycles of VAC (vincristine, dactinomycin, and cyclophosphamide) and radiation therapy (5120 cGy). Her visual acuity and ocular motility were intact 1 year after treatment. MRI revealed complete regression of the previous tumor, mucosal wall thickening, and sinusitis (Fig. 3). A positron emission tomography scan showed no distant metastases. There was no local recurrence of tumors for a total follow-up period of 1.5 years; afterwards, loss of follow-up was occurred.

## 3 Discussion

Adult rhabdomyosarcoma rarely presents in adults. Orbital involvement is one of the most favorable factors in children, and the 5-year survival rate is >90%.^[5,6]^ Because of its rarity, no study has reported outcome statistics or an established therapeutic method. The bad prognosis for adults with orbital involvement is the difficulty excising the mass, the histopathological subtype, metastasis, and poor tolerance to intensive chemotherapy.^[7]^ Tumors in the paranasal sinus, nasopharynx, infratemporal palatine fossa, or orbit more often spread into surrounding tissues. These regions have a 35% incidence of meningeal recurrence.^[8]^ The alveolar subtype is more frequent in adults and the embryonic subtype is rare. The alveolar subtype has a higher metastatic rate than that of the embryonic or pleomorphic subtypes.^[2,9,10]^ More than 60% of adult patients have regional or distant metastases at the initial diagnosis, and most rhabdomyosarcoma cases are classified as IRSG group 3.^[3]^ Therefore, the prognosis is poor because of impossible complete excision.

The treatment protocol for adults with rhabdomyosarcoma has not been established. Surgery is performed in most cases, and chemotherapy and radiotherapy are used as adjuncts following the pediatric treatment protocol. Surgery is used to debulk, reduce the mass effect on the optic nerve, and improve ocular motility.^[11]^ A combination of chemotherapy and radiation therapy increases the local remission rate and decreases the metastatic rate. The conventional chemotherapeutic agents used are vincristine, dactinomycin, and cyclophosphamide. Darren et al^[8]^ suggested that 50 to 56 Gy was adequate for patients with completely resected disease and a negative resection margin. We also performed debulking surgery, conventional chemotherapy, and radiation therapy. There have been 7 recent cases similar to this case. Two cases died from systemic complications associated with the invasion.^[12]^ In other 2 reports, there was no mention of therapeutic response or progression because the case was in the middle of treatment.^[13,14]^ The remaining 3 cases survived for >2 years.^[15–17]^ Two cases were treated with chemotherapy. The other 1 case was treated with surgery and chemotherapy. Prognosis is worse in maxillary rhabdomyosarcoma than in other sinus rhabdomyosarcoma because extensive local disease is usually found at the time of diagnosis, making surgical excision difficult or impossible, and because local and distal metastasis is often found when treatment is initiated. There has been no recent report of a case of maxillary rhabdomyosarcoma with orbital extension in which a remission has been confirmed through imaging for 1 year. Although the duration of follow-up is short in our case, but the value of the report is considered sufficient.

In conclusion, adult rhabdomyosarcoma is encountered very rarely in the orbit, where it is usually an aggressive malignancy with a poor prognosis. Rhabdomyosarcoma should be considered in the differential diagnosis of a rapidly growing orbital mass. Aggressive treatment, including surgery, chemotherapy, and radiation therapy can increase local remission rates and improve the prognosis.

## Author contributions

**Conceptualization:** Jin-Ho Joo.

**Data curation:** Shin-Myeong Choi.

**Investigation:** Shin-Myeong Choi.

**Supervision:** In-Ki Park, Jae-Ho Shin.

**Writing – original draft:** Jin-Ho Joo.

**Writing – review & editing:** Ji Sang Han, Jae-Ho Shin.

Jin-Ho Joo Orcid: 0000-0003-0846-6465.
