# One-year survivor of adult alveolar rhabdomyosarcoma of the maxillary sinus with orbital extension: Case report: Erratum

**DOI:** 10.1097/MD.0000000000014294

**Published:** 2019-01-25

In the article, “One-year survivor of adult alveolar rhabdomyosarcoma of the maxillary sinus with orbital extension”,^[1]^ which appeared in Volume 97, Issue 35 of *Medicine*, the authors would like to note the following:

This research was supported by the National Research Foundation of Korea (NRF) funded by the Ministry of Science, ICT & Future Planning (2017R1C1B1006736).
